# Case report of unusual cause of trigeminal neuralgia: Trigeminal neuralgia secondary to enlarged suprameatal tubercle

**DOI:** 10.1016/j.amsu.2021.102308

**Published:** 2021-04-15

**Authors:** Bilal Ibrahim, Baha'eddin A. Muhsen, Edinson Najera, Hamid Borghei-Razavi, Badih Adada

**Affiliations:** Department of Neurosurgery, Braathen Center, Cleveland Clinic Florida, Weston, FL, USA

**Keywords:** Case report, Trigeminal neuralgia, Enlarged suprameatal tubercle, Trigeminal nerve decompression

## Abstract

**Background:**

Classic trigeminal neuralgia (TN) is caused by vessels compressing the trigeminal nerve root entry zone. The cause is usually impingement of the superior cerebellar artery, anterior inferior cerebellar artery, or a vein. Other rare causes have been reported including aneurysmal compression, skull base tumors, and vascular malformations. An enlarged suprameatal tubercle (EST) as a cause of TN has not yet been described.

**Case presentation:**

We report the first case of 37 year old female patient presented with severe TN involving the three branches of trigeminal nerve who failed medical treatment and underwent multiple balloon compression for left TN with minimal improvement. The severity of pain was assessed using Barrow Neurological Institute (BNI) pain intensity score. Patient had brain MRI with CISS sequence and CT scan for the brain. After careful revision of her imaging studies, patient found to have prominent and heavily calcified left supra meatal tubercle. Her preoperative BNI score was 5.

Patient had left retrosigmoid craniotomy and drilling of left suprameatal tubercle. No other structures were seen in contact with left trigeminal nerve root entry zone. Patient had significant improvement on her pain, postoperative BNI score was 1 until the last follow-up 4 years.

**Conclusion:**

EST is a rare cause of TGN and should be suspected as the offending compressing structure when no other causes seen on imaging studies.

## Highlights

• Enlarged suprameatal tubercle as a rare cause of trigeminal neuralgia.
• Trigeminal neuralgia due to bony compression have to be ruled out in obscured cases.
• Drilling of suprameatal tubercle to decompress trigeminal nerve root entry zone.

## 1 Introduction

Trigeminal neuralgia (TN) is most commonly caused by compression of trigeminal nerve at root entry zone by tortious superior cerebellar artery [1,2]. Other rare causes have been reported including tumors, (such as petroclival meningiomas, vestibular schwannomas, etc.) or enlarged veins [2,7].

The pain severity and treatment response is usually assessed by BNI pain intensity score (Table 1). Treatment typically starts with medical management with carbamazepine or oxcarbazepine. Other medications such as lamotrigine, gabapentin, botulinum toxin type A, pregabalin, baclofen, and phenytoin may be used either alone or as add-on therapy [17].

**Table 1:** Barrow neurological institute (BNI) pain intensity score.

Score	Pain description
I	No pain, no medications required.
II	Occasional pain, no medications required.
III	Some pain, adequately controlled by medications.
IV	Some pain, not adequately controlled by medications.
V	Severe pain or no pain relief with medications.

Surgery is offered for patients who are refractory to medical therapy [17]. Microvascular decompression of the trigeminal nerve is considered the gold standard procedure and has the longest pain-free status compared to other neuroablative procedures (balloon compression, glycerol injection, gamma-knife surgery, etc.), specially for typical trigeminal neuralgia pain [3,4,6,17].

Bony abnormalities, as petrous bone deformities or basilar impression, causing compression over trigeminal nerve or nucleus have been reported previously [[8], [9], [10]]. However, EST as a cause of TN has not been reported yet. We present the first case of TN caused by EST in a patient who had complete pain relief after drilling the suprameatal tubercle. This work has been reported in line with the SCARE criteria [18].

### 1.1 Case description

**Fig. 1:**
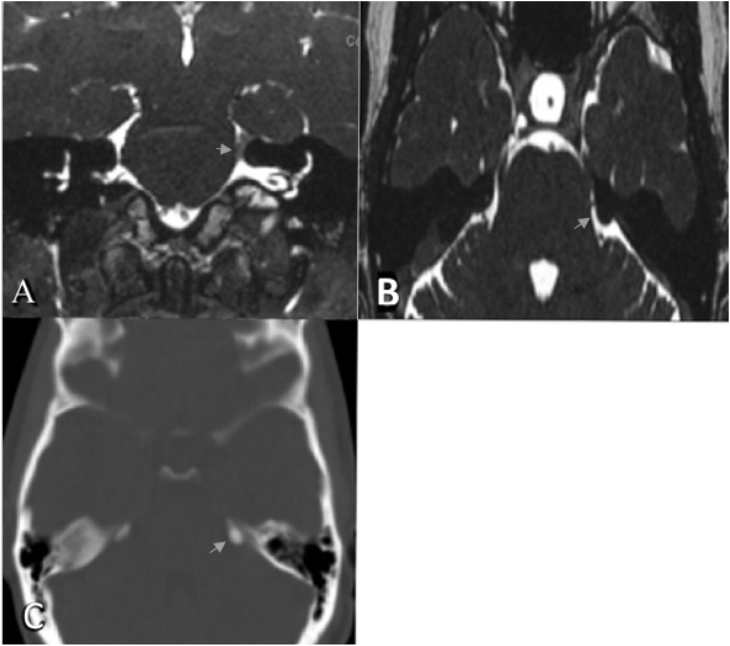
A) and B): Preoperative MRI brain axial and coronal T2 CISS showing left EST (arrows) in close proximity to trigeminal nerve REZ. C) CT scan brain axial cut bone window showing left prominent suprameatal tubercle (arrow).

A 37-year-old, right-handed, medically free, female patient presented to the clinic with left sided TN refractory to medical therapy. Her pain was in the distribution of the three branches of the trigeminal nerve and electric in nature. She had daily pain despite multiple medications including carbamazepine, baclofen, and pregabalin. On presentation, her BNI Pain Intensity Score was 5, physical examination, including cranial nerves examination, was unremarkable. Typically she had acceptable pain relief from medication but then the pain would recur. Magnetic resonance imaging (MRI) of the brain with constructive interference in steady state (CISS) sequence did not show any abnormal vascular compression, however, a prominent left suprameatle tubercle was noted. All the options were discussed with the patient and she agreed to proceed with balloon compression. The patient had an improvement of her symptoms for 6 months after the balloon compression before the pain recurred. She then underwent a second balloon compression which gave her a relief for 14 months before the pain recurred with more intensity. Therefore, a third balloon compression was performed but the patient continued to have pain in the mandibular distribution (V3) of the trigeminal nerve with minimal pain relief. Another brain MRI was obtained which showed no vascular loops pressing on trigeminal nerve, however, the EST was visualized compressing the upper aspect of the nerve with impingement of the superior part of the nerve (Fig. 1. A, B). A CT brain was completed and showed enlarged and heavily calcified suprameatal tubercle (Fig. 1. C).

The EST as a possible cause of TGN and surgical intervention were discussed with patient extensively, including benefits of surgery and possible complications. She decided to proceed for the surgery.

Other than the aforementioned medications, patient was not on any other prescribed drugs.

### 1.2 Operative procedure and results

**Fig. 2:**
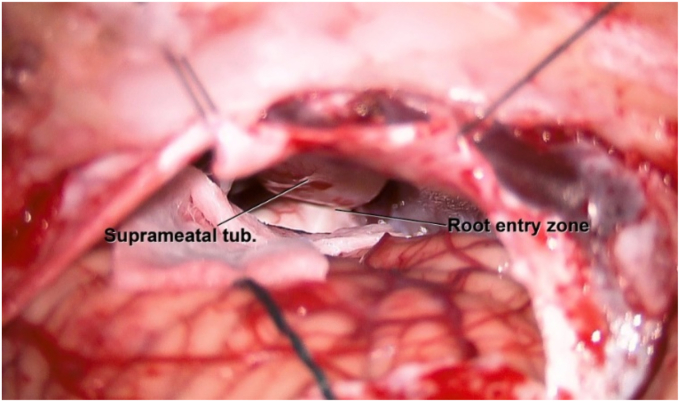
Intraoperative microscopic view showing prominent and calcified suprameatal tubercle covering the cisternal segment of trigeminal nerve.

**Fig. 3:**
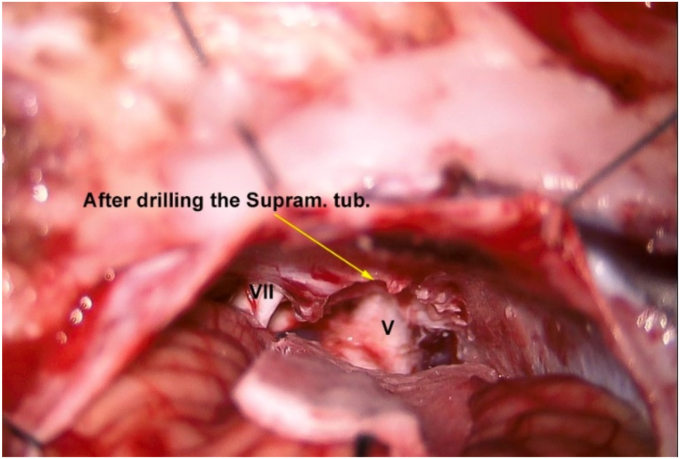
The entire length of trigeminal nerve can be seen after drilling of EST. Note the pressure mark over the nerve caused by EST compression.

The operation done by the most senior author (B.A). Patient positioned supine with head elevated thirty degree tilted to the right side. Patient's left shoulder was elevated with towel rolls to achieve the appropriate lateral position of the head. Retro-auricular C-shaped skin incision was done. Musculocutaneous flap was retracted anteriorly. Transverse sigmoid junction location approximated using superficial bony landmarks and burr hole created over the junction. Craniotomy was done and bone flap elevated after careful dissection of dura underlying the bone flap. Dura was incised and cerebrospinal fluid drained to allow cerebellum relaxation. With dynamic cerebellum retraction using suction tube and bipolar, EST was encountered concealing about two thirds of the lateral cisternal segment of trigeminal nerve (Fig. 2). Seventh and eight cranial nerve complex identified. Enlarged suprameatal tubercle was exposed. Dura over the tubercle was incised and dissected all around. EST was drilled carefully using low profile drill in the direction of Meckel's cave. Superior cerebellar artery visualized and was not in contact with the nerve. After complete removal of the EST, impingement mark was noted over the nerve before entering Meckel's cave (Fig. 3). Bone wax was applied over the stump of the drilled tubercle. Adequate hemostasis and watertight dural closure were performed. Patient had excellent post-operative recovery and no intra- or post-operative complications.

**Fig. 4:**
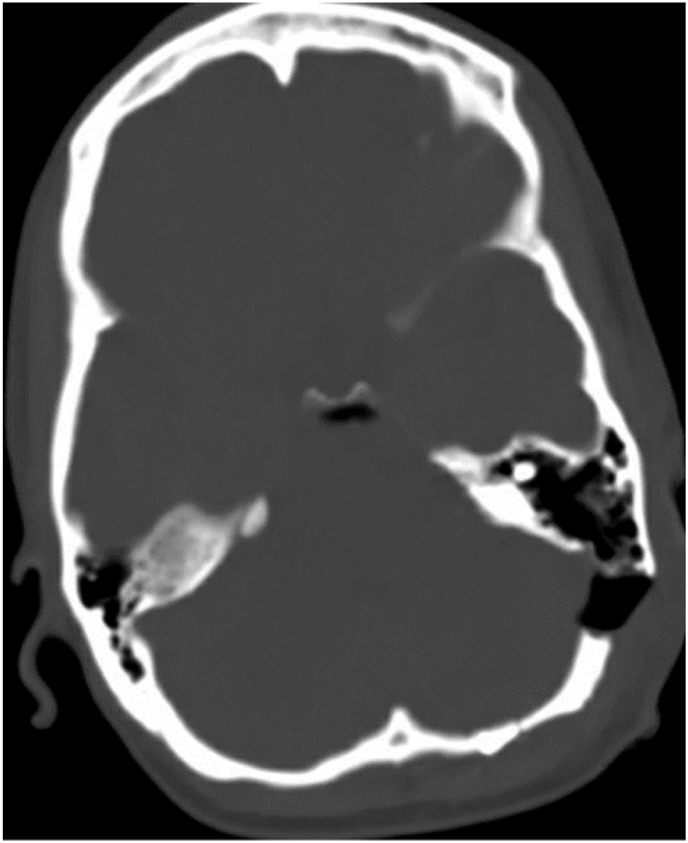
Post-operative CT-scan bone window, showing complete drilling of left suprameatal tubercle.

Post-operative brain CT scan was done which showed the suprameatal tubercle flush with the posterior aspect of petrous bone (Fig. 4). Patient had complete relief of her symptoms in the immediate post-operative period (BNI Pain intensity score 1). Medication was tapered three months after surgery and patient continued to report absence of her trigeminal neuralgia pain. Last follow-up with the patient was 1 year after the surgery in the neurology clinic and patient had no TN pain recurrence.

## 2 Discussion

Trigeminal neuralgia, also known as tic douloureux, is a chronic, neuropathic, unilateral facial pain over the distribution of one or more braches trigeminal nerve. The annual incidence is about 4/100,000. In the revised ICHD-III (International Classification of Headache Disorders, 3rd edition, beta version), TN is classified into: classical TN (without or with concomitant persistent facial pain), and painful trigeminal neuropathy which is related to tumors, multiple sclerosis plaque, herpes zoster, or trauma [5].

The most common cause of TN is compression of trigeminal nerve at root entry zone by tortious superior cerebellar artery [1,2], however, other causes such as venous compression, persistent primitive trigeminal artery, or skull base tumors have been reported [2,7,11].

Bony structures or pathologies could be the offending pathologies that cause TN. Mata-Gómez et al. described a case of TN caused by trigeminal nerve compression by endostosis of petrous bone in which the pain disappeared after endostosis drilling [9]. Hirata et al. reported a case of TN caused by petrous apex bone deformity causing compression of the cisternal segment of the trigeminal nerve for which they did anterior transpetrosal approach with drilling of petrous apex [10]. In the both reported cases, the symptoms of TN were completely resolved (Table 2 under this paragraph). Other rare causes of TN by bony pathologies include basilar impression and Paget's disease [12].

**Table 2:** Patients characteristics and postoperative results.

Study	Age	Sex	Pain distribution	Compressing pathology	Surgical approach	Other compressing structures	Response to surgery	F/U duration	Symptoms recurrence
Hirata et al.	43 year	M	L.t V2/V3	Petrous bone deformity	ATPA	SCA	Complete symptoms resolution	U/N	U/N
Mata-Gómez	44 year	FM	R.t V1/V2/V3	Petrous bone endostosis	Retrosigmoid craniotomy	None	Complete symptoms resolution	1 Year	None
Our case	37 year	FM	L.t V1/V2/V3	EST	Retrosigmoid craniotomy	None	Complete symtoms resulation	1 year	None

ATPA: Anterior transpetrosal approach, EST: Enlarged suprameatal tubercle, FM: Female, L.t: Left, M: Male, R.t: Right, U/N: Unknown, V1: Ophthalmic nerve, V2: Maxillary nerve, V3: Mandibular nerve.

Suprameatal tubercle is a part of posterior surface of temporal bone, which is the largest bony protrusion encountered during retrosigmoid approach above the upper margin of internal auditory meatus [13]. Drilling of suprameatal tubercle give access to Meckel's cave and posterior part of middle cranial fossa [13]. However, drilling of supameatal tubercle maybe necessary in cases of microvascular decompression of trigeminal nerve if heavily calcified and enlarged tubercle encountered to expose the entire length of trigeminal nerve, especially when the offending vessel is not visualized under the enlarged tubercle [[14], [15], [16]]. Inoue et al. reported EST in 48 (10.4%) of 461 patients treated by microvascular decompression for TN and 8 patients had resection of the enlarged tubercle (7 operated via retrosigmoid approach and 1 via anterior transpetrosal approach). In all of his cases, he encountered a vascular compression or impingement after drilling the EST [16].

In this case report, no vascular loops were seen in contact with the trigeminal nerve. EST was the only structure seen causing compression over the nerve. Generally, resection of EST should be done in cases where the neurovascular compression could not be seen or when microvascular decompression could not be achieved adequately due to obstructed view by the enlarged tubercle. Rarely, the EST could be the cause of compression over the nerve as in our presenting case.

## 3 Conclusion

We are presenting the first case that EST compression on the trigeminal nerve was the only cause of TN. Careful review of pre-operative imaging is necessary to rule out this rare cause of TN when no vascular loops or tumors causing compression over trigeminal nerve.

## Consent

Written informed consent was obtained from the patient for publication of this case report and accompanying images. A copy of the written consent is available for review by the Editor-in-Chief of this journal on request.

## Provenance and peer review

Not commissioned, externally peer-reviewed.

## Ethical approval

Not applicable as the published work is case report.

## Funding

This work did not receive any specific grant from funding agencies in the public, commercial or not-for-profit sectors.

## Author contribution

B.I: Data collection, writing the paper, literature review.

B.M: Data collection, writing the paper.

E.N: Images and tables editing, review of manuscript.

H.B.R: Review of manuscript, study design.

B.A: Review of manuscript, study design, data interpretation.

## Guarantor

B.A.

## Declaration of competing interest

There are no conflicts of interest for the corresponding or co-authors.
